# A standardized approach to test missense *PNPLA1* rare genetic variants of uncertain significance in epidermal differentiation disorders

**DOI:** 10.1016/j.xjidi.2025.100442

**Published:** 2025-12-13

**Authors:** Nuria Pell, Pauline Bernard, Séverine Courrech, Lukas Opalka, Aina Millán-Sánchez, Elise Levy, Séverine Garnier, Cyrielle Clément, Pauline Le Faouder, Katerina Vávrová, Olga López, Justine Bertrand-Michel, Corinne Leprince, Isabelle Fourquaux, José-Enrique Mejia, Jean-Christophe Pagès, Juliette Mazereeuw-Hautier, Nathalie Jonca

**Affiliations:** 1 Toulouse Institute for Infectious and Inflammatory Diseases (INFINITy), Toulouse University, CNRS, Inserm, Toulouse, France; 2 PEFAVar and Department of Cell Biology and Cytology, Federative Institute of Biology, Purpan Hospital, University Hospital, Toulouse, France; 3 Reference Center for Rare Skin Diseases, Dermatology Department, CHU Toulouse, Toulouse University, Toulouse, France; 4 Faculty of Pharmacy, Charles University, Hradec Králové, Czech Republic; 5 Institute for Advanced Chemistry of Catalonia (IQAC-CSIC), Barcelona, Spain; 6 MetaboHUB-MetaToul, National Infrastructure of Metabolomics and Fluxomics, Toulouse, France; 7 I2MC, Inserm, Toulouse University, Toulouse, France; 8 Centre de Microscopie Electronique Appliquée à la Biologie, Toulouse University, Toulouse, France; 9 RESTORE Institute, UMR INSERM 1301 CNRS U5070, UT, ENVT, EFS, Toulouse, France

**Keywords:** Functional genetics, Genodermatosis, Human epidermal equivalents, Ichthyosis, Skin barrier

## Abstract

Congenital ichthyoses, now renamed epidermal differentiation disorders (EDDs) (syndromic EDD or nonsyndromic EDD), are rare, disabling conditions caused by sequence variations in epidermal barrier genes. However, 5–10% of variants, called “variants of uncertain significance” (VUS), remain uncharacterized, and their pathogenicity is not demonstrated. We developed an approach for classifying VUS in nonsyndromic EDD associated with variant in *PNPLA1*. We generated *PNPLA1*-knockout human keratinocytes. PNPLA1, encoded by a missense VUS or by the reference coding sequence, was expressed after lentiviral transduction in the *PNPLA1*-knockout cells. Transduced cells were used to produce human epidermal equivalents, and the functionality of the normal or VUS-encoded proteins was evaluated. Compared with *PNPLA1*-knockout human epidermal equivalents re-expressing normal PNPLA1, *PNPLA1*-knockout human epidermal equivalents showed disrupted synthesis of ω-O-acylceramide, the normal product of PNPLA1, as well as abnormal vesicle-like structures and immature cornified envelopes, characteristic of the epidermis of patients with *PNPLA1* variants. Human epidermal equivalents expressing the *PNPLA1* VUS showed similar abnormalities, consistent with an impaired PNPLA1 function. This work demonstrated a feasible strategy to help reclassifying missense VUS, which can be extended to other EDD-related genes. Although further efforts are needed to translate this approach into clinical practice and help overcome current diagnostic limitations, such models are valuable tools for pathophysiological and preclinical research on EDDs.

## Introduction

Epidermal differentiation disorders (EDDs) (syndromic EDD [sEDD] and nonsyndromic EDD [nEDD]) are inherited skin conditions affecting the epidermis (Akiyama et al, 2025; Hernández-Martín et al, 2025; Paller et al, 2025; Sprecher et al, 2025). They include a group of rare genetic disorders formerly known as autosomal recessive congenital ichthyoses, characterized by scaling, skin thickening, and often erythema and skin fragility. These diseases are usually severe and have a significant impact on patient QOL (Gutiérrez-Cerrajero et al, 2023; Oji et al, 2010). Resulting from sequence variations in skin barrier function genes, they have a high genetic heterogeneity, with >60 genes identified so far. Missense variants represent the most significant genetic alterations (Akiyama, 2010; Farasat et al, 2008; Richard, 2004; Wertheim-Tysarowska et al, 2024; Youssefian et al, 2019; Zimmer et al, 2017).

Genetic variants are classified on the basis of their impact on gene function and clinical presentation, considering population frequency, nature of the genetic change, in silico predictions, and functional or clinical evidences. They are categorized as "pathogenic," "likely pathogenic," "benign," "likely benign," and "variants of uncertain significance" (VUSs) (Richard, 2004; Zhang et al, 2020a). VUSs lack sufficient or consistent data on pathogenicity. In particular, for missense variants, in vivo or in vitro functional studies may prove necessary to confirm effects suggested by in silico prediction tools and avoid classification of the variant as a VUS.

VUS in patients showing monogenic diseases complicates genetic counseling and clinical decision making. Moreover, it can hinder the functional characterization of new or rare variants, impeding the progress in understanding the pathophysiology. The incidence of VUS in nEDD is relatively high. In a large cohort of 265 Polish patients with nEDD or sEDD, among the 32 newly identified variants, 17 were VUSs (Wertheim-Tysarowska et al, 2024). In an Iranian cohort of 112 consanguineous families with genetically diagnosed nEDD, 7 families were identified with VUSs (6.25%) (Youssefian et al, 2019). According to data collected from our University Hospital, within a cohort of 122 unrelated patients with nEDD or sEDD genotyped between 2012 and 2023, 22 (18%) were harboring VUSs. In the 3 cohorts, most VUSs were missense variations.

In this study, we report a standardized approach for classifying missense VUSs. We used the N/TERT-2G immortalized keratinocyte cell line to produce genetically modified human epidermal equivalents (HEEs) and tested whether a variant of interest was able to rescue the genetic deficiency in HEEs. We demonstrated the feasibility of this approach by successfully analyzing 3 missense VUSs in the nEDD-involved gene *PNPLA1*. We anticipate that it can be extended to help classifying missense VUSs in a broader range of autosomal recessive nEDD- or sEDD-related genes.

## Results

### Identification of *PNPLA1* VUSs in patients with nEDD

**Figure 1:**
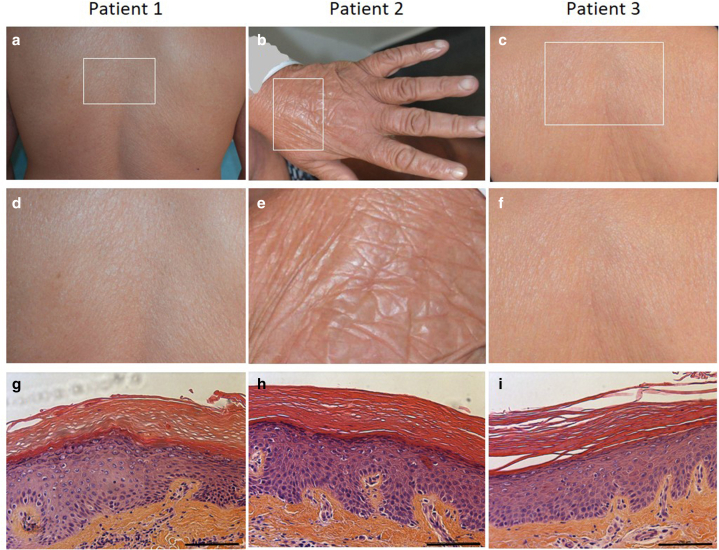
**Clinical and histological features of patients carrying *PNPLA1* variants.** The skin of (**a, d, g**) patients 1, (**b, e, h**) patients 2, and (**c, f, i**) patients 3 were examined. (**a–c**) Back skin in patients 1 and 3 and skin of the dorsal hand of patient 2. (**d−f**) Enlargement of corresponding surrounded area in **a–c**, respectively. (**g−i**) H&E-stained skin sections (bars = 100 μm). Full informed written consent was obtained from the patient, including consent to the publication of images.

Three unrelated patients (P1–P3) with European, nonconsanguineous parents exhibited typical nEDD features. All were born with a collodion phenotype and later developed mild EDD with moderate scaling, slight erythema, pruritus, and ectropion in 1 case (P2). Skin biopsies showed acanthosis and marked hyperkeratosis (Table 1 and Figure 1).

**Table 1:** Clinical and Genetic Data from the Patients

Patient n°	Sex	Age at Evaluation, y	Consanguinity of Proband's Parents	Ethnicity	Collodion Phenotype at Birth	Ectropion	Erythroderma	Pruritus	*PNPLA1* Nucleotide Variant^1^	PNPLA1 Amino Acid Change^2^	Target VUS
1	M	36	No	European	Yes	No	Yes (3/10)	Yes (1/10)	c.[**149C>A**];[488C>T]	p.[**Ala50Glu**];[Pro163Leu]	VUS.149
2	F	54	No	European	Yes	Yes	Yes (5/10)	Yes (8/10)	c.[**485C>G**];[**485C>G**]	p.[**Pro162Arg**];[**Pro162Arg**]	VUS.485
3	F	62	No	European	Yes	No	Yes (4/10)	Yes (5/10)	c.[**536A>G**];[736C>T]	p.[**Gln179Arg**];[Arg246Ter]	VUS.536

Abbreviations: F, female; M, male; VUS, variant of uncertain significance.

1 Reference sequence *PNPLA1*, NM_001374623.1; VUS are in bold.

2 Reference sequence PNPLA1: NP_001361552.1; VUS are in bold.

Sequence variation screening by next-generation sequencing identified in each of the 3 patients genetic variations in the nEDD-related gene *PNPLA1*. This was confirmed by bidirectional Sanger sequencing (Table 1). No disease-causing variants were detected in other genes involved in EDD present in our custom panel of 108 genes. Three *PNPLA1* missense variants lacked sufficient evidence for pathogenicity and were considered VUSs (Table 2). The first, c.149C>A (p.(Ala50Glu)), was identified at the heterozygous state in P1. It affected a conserved amino acid in mammals, located in the patatin*-*like domain of the protein. Although it was reported in the ClinVar database as likely pathogenic, in silico predictions of its effect on the protein function were divergent (SIFT, PolyPhen-2, Mutation Taster). The second VUS, c.485C>G (p.(Pro162Arg)), was identified at the homozygous (or hemizygous) state in P2. Although in silico tools predicted that the substitution of this highly conserved proline by an arginine within the conserved patatin*-*like domain was deleterious, this variant has never been reported in the literature or genomic databases. P3 was a compound heterozygote for a class 4 nonsense variant and the third identified VUS, c.536A>G (p.(Gln179Arg)). This VUS affected a highly conserved amino acid in mammals, also located in the Patatin*-*like domain of *PNPLA1*. It was absent from genomic databases, although it has previously been reported in a compound heterozygous state in a patient with nEDD (Zimmer et al, 2017).

**Table 2:** Genetic Variants Identified in Patients

Variant at cDNA Level^1^	Variant at Protein Level^2^	Exon	Type of Variation	Protein Domain	Allele Frequency (GnomAD)	dbSNP	PolyPhen 2.1^3^	SIFT^4^	Mutation Taster^5^	ClinVar	Pathogenicity Classification	References
c.149C>A	p.Ala50Glu	1	Missense	Patatin-like	6.44e-7	rs533584507	Probably damaging (score: 0.998)	Tolerated (score: 0.08)	Deleterious	ID:633797	Class 3	Youssefian et al (2019)
c.485C>G	p.Pro162Arg	3	Missense	Patatin-like	Absent	Absent	Probably damaging (score: 1.000)	Deleterious (score: 0.00)	Deleterious	absent	Class 3	This report
c.488C>T	p.Pro163Leu	3	Missense	Patatin-like	Absent	rs777658285	Probably damaging (score: 1.000)	Deleterious (score: 0.00)	Deleterious	ID:2579650	Class 4	Vahidnezhad et al (2017)
c.536A>G	p.Gln179Arg	4	Missense	Patatin-like	Absent	Absent	Probably damaging (score: 0.999)	Deleterious (score: 0.00)	Deleterious	absent	Class 3	Akiyama (2010)
c.736C>T	p.Arg246Ter	5	Nonsense	None	1.05e-5	rs777268917	NA	NA	NA	ID:2445879	Class 4	Lima Cunha et al (2021)

Abbreviations: ID, identification; NA, not available.

1 Reference sequence *PNPLA1*, NM_001374623.1.

2 Reference sequence PNPLA1: NP_001361552.1.

3 http://genetics.bwh.harvard.edu/pph2/index.shtml.

4 https://sift.bii.a-star.edu.sg/www/SIFT_aligned_seqs_submit.html.

5 https://www.mutationtaster.org/ChrPos.html.

### Functional testing strategy and generation of 3-dimensional *PNPLA1*-knockout models of *PNPLA1* nEDD

**Figure 2:**
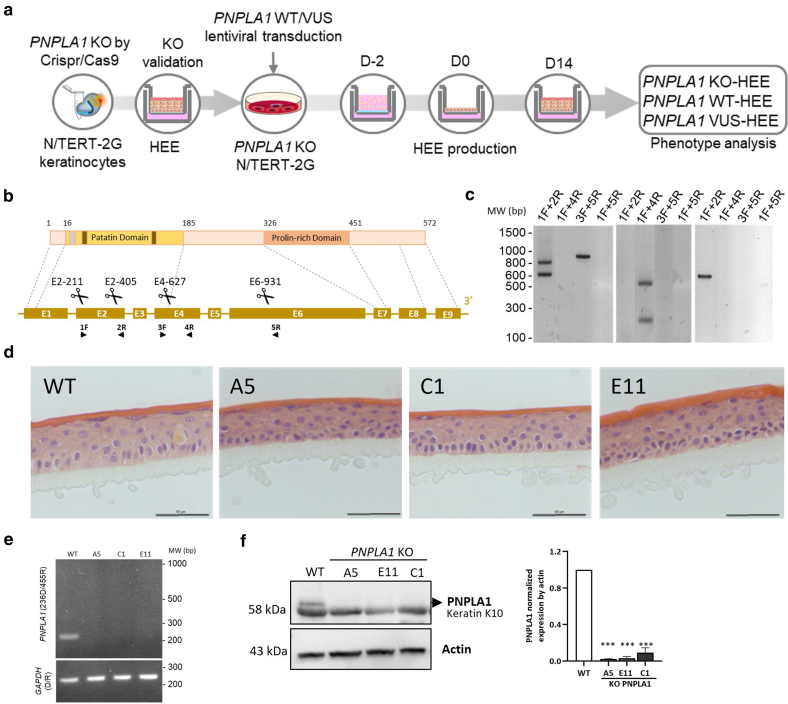
**VUS testing strategy and generation of *PNPLA1*-KO model.** (**a**) Strategy to generate *PNPLA1*-KO N/TERT-2G HEEs and HEEs expressing the WT- or VUS-encoded PNPLA1. (**b**) Diagram of PNPLA1 protein (top) and gene (bottom). Amino acid numbers and protein domains (patatin-domain: yellow box; oxyanion hole: gray stripe; catalytic dyad: brown stripe; prolin-rich domain: orange box) are provided. Exons (blocks), introns (line), and sites targeted by sgRNAs are shown. Arrowheads: position of the primers used in **c**. (**c**) PCR screening of large genomic rearrangements in selected N/TERT-2G clones after *PNPLA1* genome edition, using the indicated primer pairs. (**d**) H&E images of WT and *PNPLA1*-KO HEEs. Bar = 50 μm. (**e**) RT-PCR analysis of RNA from the HEE samples. *GAPDH* was used as the reference gene. (**f**) Western blot analysis of PNPLA1 expression in HEE samples (left panel). Quantification of *PNPLA1* expression was normalized by actin (right panel). Data are presented as mean ± SEM, with 1-way ANOVA test, ∗∗∗*P* < .001. MW denotes molecular weight, bp denotes base pair, and kDa denotes kilodalton. D, day; HEE, human epidermal equivalent; KO, knockout; sgRNA, single-guide RNA; VUS, variant of uncertain significance; WT, wild type.

Our experimental strategy to test the pathogenicity of missense *PNPLA1* VUS required the generation of *PNPLA1* CRISPR/Cas9-knockout (KO) N/TERT-2G immortalized keratinocytes. The normal (referred to as the "wild type" [WT] for convenience in the remaining parts of this paper) or VUS-encoded PNPLA1 proteins were secondarily expressed in the KO cell line by lentiviral transduction. HEEs were then generated using transduced and control cells, and their phenotype was analyzed (Figure 2a).

**Figure 3:**
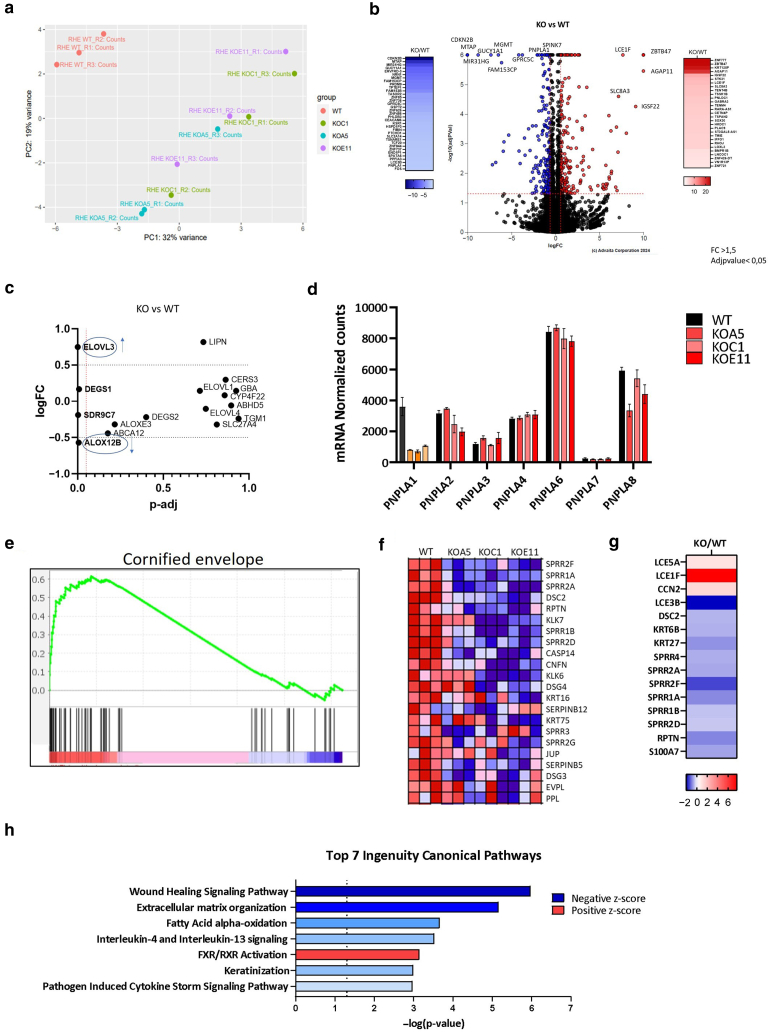
**RNA-sequencing analysis of WT- and *PNPLA1*-KO N/TERT-2G HEEs.** (**a**) PCA of WT and *PNPLA1-*KO samples. Each dot represents 1 HEE sample. (**b**) Volcano plot of upregulated (red) and downregulated (blue) transcripts in *PNPLA1-*KO N/TERT-2G HEEs compared with those in WT N/TERT-2G HEEs. (**c**) Analysis of ceramide synthesis–involved genes. (**d**) Analysis of genes of the *PNPLA* family. mRNA normalized counts were extracted from RNA-sequencing analysis (mean ± SEM, n = 3 HEEs per condition). (**e**) GOCC gene set enrichment analysis plot. (**f**) Heat map showing CE involved genes (GOCC) upregulated (red) or downregulated (blue) in the *PNPLA1*-KO HEE samples compared with those in WT HEE samples (n = 3 samples per condition). (**g**) Significantly upregulated (red) or downregulated (blue) genes involved in keratinization, CE, and keratinocyte formation. GSEA was performed on pooled data from the 3 *PNPLA1*-KO compared with those from WT samples. (**h**) Top 7 upregulated (red) and downregulated (blue) enriched pathways using Ingenuity Pathway Analysis. CE, cornified envelope; GOCC, Gene Ontology Cellular Component; GSEA, gene set enrichment analysis; HEE, human epidermal equivalent; KO, knockout; NES, normalized enrichment score; PCA, principal component analysis; WT, wild type.

Three CRISPR/cas9 *PNPLA1*-KO single-cell clones, namely A5, C1, and E11, were selected after genome editing targeting *PNPLA1* (Figure 2b). Each bore distinct genomic rearrangements in *PNPLA1*, all affecting the coding sequence (Table 3 and Figure 2c). Because *PNPLA1* is expressed in late differentiated keratinocytes, *PNPLA1* loss of expression was checked in HEEs generated using WT or *PNPLA1-*KO N/TERT-2G clones. Histologically, HEEs did not show any morphological differences according to their genotypes (Figure 2d). RT-PCR (Figure 2e) and western blot analyses (Figure 2f) confirmed the absence of expression of *PNPLA1* in KO HEEs. *PNPLA1* KO in HEEs was further assessed by RNA-sequencing analysis. Principal component analysis showed different sample clustering for *PNPLA1* KO and WT (Figure 3a). Differences in gene expression in *PNPLA1*-KO versus WT N/TERT-2G HEEs were modest overall. Among the 508 differentially expressed genes (fold change > 1.5, adjusted *P* < .05), 295 were significantly upregulated, and 213 were downregulated (Figure 3b). Except *PNPLA1*, the expression levels of genes involved in the EO-ceramide (EO-Cer) metabolism did not differ greatly in *PNPLA1*-KO N/TERT-2G HEEs compared with that in WT N/TERT-2G HEEs. Notably, only *ALOX12B* and *ELOVL3* were differently expressed in the absence of PNPLA1 (Figure 3c). Moreover, the lack of significant difference in the expression of other PNPLA family members supported the hypothesis that there is no compensatory mechanism (Figure 3d). Gene set enrichment analysis also found enrichment in genes of the cornified envelopes (CEs) cellular compartment (Figure 3e). Among them, several *SPRR* family members were greatly downregulated in the *PNPLA1*-KO N/TERT-2G HEEs, together with keratin 6B gene *K6B* and keratin 27 gene *K27*, whereas *LCE5A* and *LCE1F* were upregulated (Figure 3f and g). Ingenuity pathway analysis identified wound healing signaling, extracellular matrix organization, fatty acid oxidation, and keratinization among the top 7 downregulated enriched pathways. On the contrary, FXR/RXR activation was significantly positively enriched (Figure 3h). We could not suspect off-target effect by screening RNA-sequencing results obtained from the 3 HEEs generated with any of the 3 *PNPLA1*-KO clones.

**Table 3:** Genomic Information on Isolated *PNPLA1*-KO N/TERT-2G Clones A5, C1, and E11

Clones	Genomic Rearrangements and Location^1^	Zygosity	Predicted Protein^2^	Protein Expression
A5	c.[228_405del;243_421inv;621_972del];[228_420del;621_972del]	Heterozygous compound	p.Val77delinsGlyArgGluLeu∗ (allele 1) p.Asn76Lysfs∗28 (allele 2)	No
C1	c.[214_714+47del];[219-630del_c.228-420inv]	Heterozygous compound	p.Val74Thrfs∗1 (allele 1) p.Leu72Argfs∗36 (allele 2)	No
E11	c.[228_420del];[228_420del]	Homozygous	p.Asn76Lysfs∗28	No

Abbreviation: KO, knockout.

1 Reference sequence *PNPLA1*, NM_001374623.1.

2 Predicted from the most upstream genomic rearrangement occurring in the coding sequence.

### PNPLA1 loss of function affects EO-Cer synthesis and stratum corneum lipid organization in HEEs

**Figure 4:**
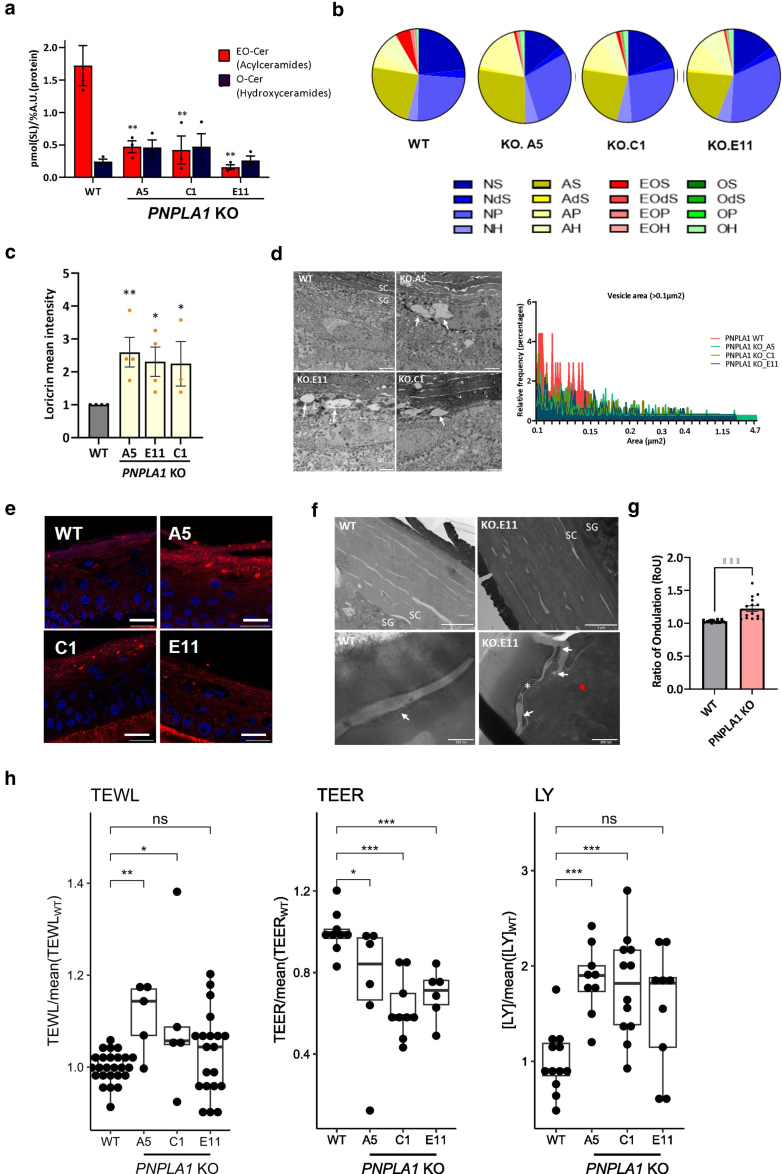
**Functional validation of *PNPLA1*-KO 3D models.** (**a**) EO-Cers and O-Cer content analysis in WT and *PNPLA1*-KO HEEs (n = 4 HEEs per condition). Data are presented as mean ± SEM, with 2-way ANOVA test, ∗∗*P* < .01 and ∗∗∗*P* < .001. (**b**) Pie chart representation of the ceramide species distribution in WT and *PNPLA1*-KO HEEs (n = 3 HEEs per condition). (**c**) Loricrin intensity in CEs extracted from the HEEs (n = 4 HEEs per condition). Data are presented as mean ± SEM, with Kruskal–Wallis test, ∗*P* < .05 and ∗∗*P* < .01. (**d**) TEM images of the SG/SC interface from the HEEs (left panel). Bar = 2 μm. White arrows: translucent vesicles. Relative quantification of vesicles area observed by TEM (right panel) (2 HEEs per condition, n ≥ 7 TEM images analyzed per condition) was performed. (**e**) Nile Red staining of HEE sections. Bar = 20 μm. Blue staining: nuclei. (**f**) FSTEM images of HEEs. Bar = 2 μm (upper panel), 200 nm (lower panel). Asterisk: corneodesmosomes, white arrows: nonlamellar domains, red arrow: abnormal vesicular structures of the SC. (**g**) Corneocyte RoU in *PNPLA1*-KO samples compared with those in the WT. Data are presented as mean ± SEM, with Student's *t*-test, ∗∗∗*P* < .001. (**h**) Box plot representation of the TEWL, TEER, and LY permeability (normalization by dividing each value by the mean of the WT measurements). Horizontal bars indicate median and interquartile range, with 1-way ANOVA test, ∗*P* < .05, ∗∗*P* < .01, and ∗∗∗*P* < .001. 3D, 3-dimensional; CE, cornified envelope; EO-Cer, EO-ceramide; FSTEM, freeze-substitution transmission electron microscopy; HEE, human epidermal equivalent; KO, knockout; LY, Lucifer yellow; ns, not significant; O-Cer, hydroxyceramide; RoU, ratio of undulation; SC, stratum corneum; SG, stratum granulosum; TEER, transepithelial electrical resistance; TEM, transmission electron microscopy; TEWL, transepidermal water loss; WT, wild type.

PNPLA1 is a transacylase essential for the skin barrier formation (Lulić and Katalinić, 2023). It catalyzes the biosynthesis of EO-Cers through the esterification of ω-hydroxy ceramides with linoleic acid (Ohno et al, 2017). We thus analyzed the ceramide composition in WT and *PNPLA1*-KO N/TERT-2G HEEs by liquid chromatography-tandem mass spectrometry (LC-MS/MS). EO-Cer was drastically decreased in the absence of PNPLA1 (Figure 4a and b), validating *PNPLA1* loss of function in the *PNPLA1*-KO N/TERT-2G HEEs.

In the stratum corneum (SC), EO-Cer is critical to establish the cornified lipid envelope and for the proper organization of free lipids between corneocytes in the extracellular lipid lamellae (Akiyama, 2017; Feingold and Elias, 2014; van Smeden et al, 2014). We assessed the maturity of the CEs by performing a loricrin immunostaining together with a Nile red staining on CEs purified from WT and KO *PNPLA1* N/TERT-2G HEEs. In the *PNPLA1*-KO conditions, CEs showed an increased loricrin staining intensity compared with the WT, suggesting a defective lipid coverage (Figure 4c). Transmission electron microscopy examination of WT and *PNPLA1*-KO N/TERT-2G HEEs revealed the presence of large (area > 0.15 μm^2^) translucent vesicles at the stratum granulosum/SC interface in the *PNPLA1*-KO N/TERT-2G HEEs that were absent in control HEEs (Figure 4d). Lipid staining of *PNPLA1*-KO N/TERT-2G HEEs cryosections with Nile red displayed vesicle-like structures of a similar size at the stratum granulosum/SC interface, suggesting a lipid content in these vesicles (Figure 4e). SC organization was further explored by freeze-substitution transmission electron microscopy analysis. Swollen corneocytes as well as intercellular spaces with irregular shapes and widths were specifically observed in the *PNPLA1*-KO N/TERT-2G HEE samples. Moreover, a higher number of vesicle-like structures were observed in the upper SC layers of *PNPLA1*-KO N/TERT-2G HEEs than in the WT N/TERT-2G HEEs (Figure 4f). The corneocyte ratio of undulation, a morphometric indicator of epidermal barrier disruption, showed significant differences between *PNPLA1*-KO samples and the WT (Figure 4g).

Finally, PNPLA1 loss of function on the barrier function was evaluated by measurements of transepidermal water loss, transepithelial electrical resistance, and penetration of Lucifer yellow. The epidermal barrier appeared affected in the *PNPLA1*-KO conditions, although only mildly. Indeed, we obtained inconstant statistical significance according to the evaluation method or the considered clone (Figure 4h).

Altogether, these results showed that *PNPLA1* deficiency in N/TERT-2G HEEs resulted in an abnormal lipid processing and organization, leading to slight defect in the epidermal barrier.

### Testing of the 3 *PNPLA1* VUSs

**Figure 5:**
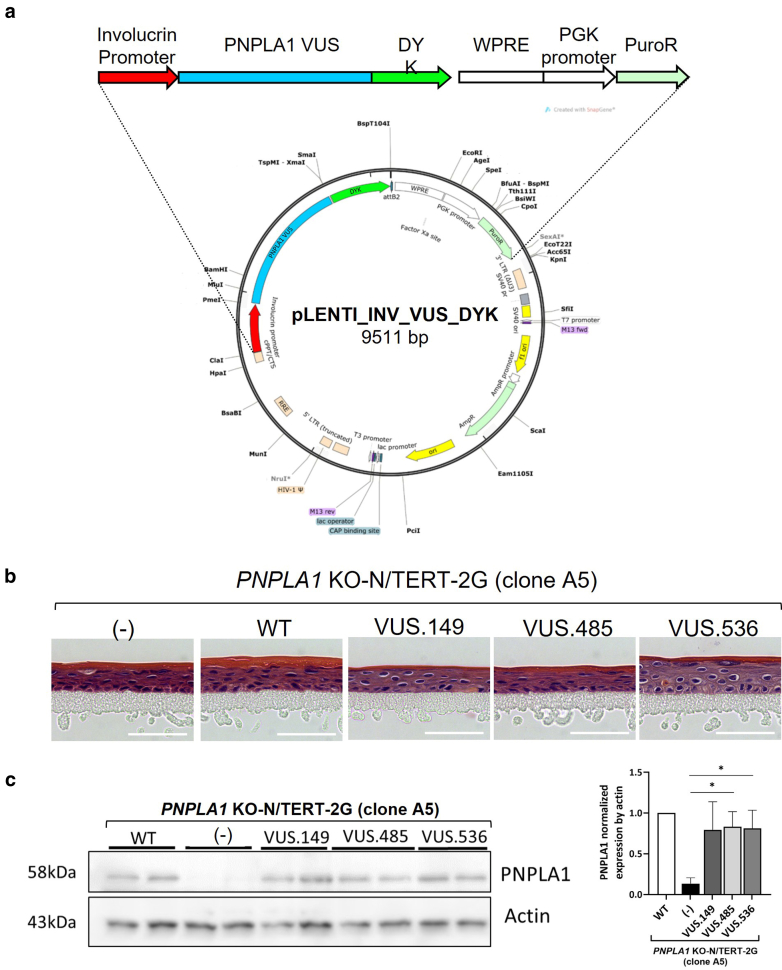
***PNPLA1* VUS transduction and testing in HEEs.** (**a**) Map of the plasmid used to transduce DYK-tagged *PNPLA1* VUS cDNAs under the control of the minimal promoter of involucrin in *PNPLA1*-KO N/TERT-2G cell clones. (**b**) H&E images of *PNPLA1*-KO HEEs (clone A5) and of HEEs generated from transduced *PNPLA1*-KO clone A5 re-expressing WT PNPLA1 or PNPLA1 encoded by VUS.149, VUS.485, or VUS.536. Bar =50 μm. (**c**) Western blot analysis of PNPLA1 expression in the indicated HEE samples (left panel). Quantification of *PNPLA1* expression was normalized by actin (n = 4 different samples per condition) (right panel). Data are presented as mean ± SEM, unpaired *t*-test for each VUS-expressing HEE versus the *PNPLA1*-KO HEE (noted [−] in the x-axis), ∗*P* < .05. HEE, human epidermal equivalent; KO, knockout; VUS, variant of uncertain significance; WT, wild type.

**Figure 6:**
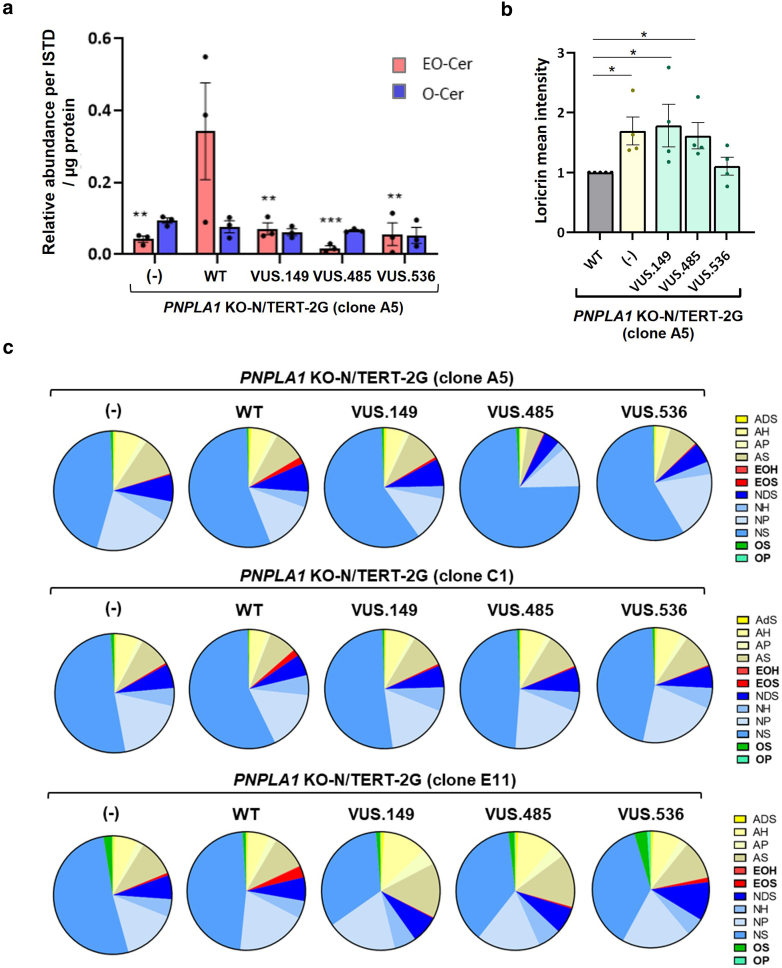
***PNPLA1* VUS testing in HEEs.** (**a**) EO-Cer and O-Cer content in the various *PNPLA1*-KO N/TERT-2G (clone A5) HEE models (n = 3 HEEs per condition). Data are presented as mean ± SEM, with 2-way ANOVA test, ∗∗*P* < .01 and ∗∗∗*P* < .001 test. (**b**) Quantification of loricrin mean intensity in CE extracted from the various *PNPLA1*-KO N/TERT-2G (clone A5) HEE models (n = 4 HEEs per condition). Data are presented as mean ± SEM, with unpaired *t*-test for each VUS-expressing HEE versus the WT HEE, ∗*P* < .05. (**c**) Pie chart representation of the ceramide species distribution in *PNPLA1*-KO N/TERT-2G HEEs (clones A5, C1, and E11) expressing WT- or VUS-encoded PNPLA1 (n = 3 HEEs per condition). CE, cornified envelope; EO-Cer, EO-ceramide; HEE, human epidermal equivalent; KO, knockout; O-Cer, hydroxyceramide; VUS, variant of uncertain significance; WT, wild type.

To test the functionality of PNPLA1 encoded by each of the 3 VUSs identified in patients, *PNPLA1*-KO clones were transduced with lentivectors carrying the WT- or the VUS-encoding *PNPLA1* sequences (namely, VUS.149, VUS.485, or VUS.536) under the involucrin promoter (Figure 5a). After selection, the transduced cells were used to generate HEEs. All had a similar morphology (Figure 5b). Western blot confirmed the expression of the VUS-encoded PNPLA1, at the same level as in *PNPLA1-*KO N/TERT-2G HEEs re-expressing WT PNPLA1 (Figure 5c). The ceramide profile showed a decreased EO-Cer content in the *PNPLA1*-KO N/TERT-2G HEEs expressing VUS.149, VUS.485, or VUS.536 in comparison with that in *PNPLA1*-KO N/TERT-2G HEEs expressing WT PNPLA1. This reduction in EO-Cer level was comparable with that seen in the *PNPLA1-*KO N/TERT-2G HEEs, suggesting that each of the 3 VUSs encoded nonfunctional proteins (Figure 6a). CE maturity assay showed an increase in loricrin signal intensity in CEs purified from *PNPLA1*-KO N/TERT-2G HEEs expressing each VUS, although below the significance threshold in the case of the VUS.536 (Figure 6b). Figure 6 illustrates experiments realized using the *PNPLA1*-KO N/TERT-2G clone A5. We obtained similar results using the clones C1 and E11 (Figure 6c). Altogether, these results show an altered function of PNPLA1 encoded by the tested VUS, providing compelling evidence supporting their pathogenicity. When integrated with existing genetic and clinical data for each patient, these results will contribute to a more accurate reclassification of these variants.

## Discussion

Our strategy for testing the functionality of *PNPLA1* missense VUSs is based on the use of genetically modified HEEs. This required first to generate and thoroughly phenotype *PNPLA1*-KO N/TERT-2G HEEs (Van Den Bogaard et al, 2021). The epidermal barrier appeared mildly impaired in the *PNPLA1*-KO N/TERT-2G HEEs compared with that in the WT N/TERT-2G HEEs. This observation is in line with the phenotype of the disease in patients and the dog model with *PNPLA1* variants, which is typically mild compared with more severe forms of EDD (Boyden et al, 2017; Diociaiuti et al, 2018; Grall et al, 2012; Pichery et al, 2017). Furthermore, *PNPLA1* loss of function was clearly confirmed in our HEE models by showing the absence of EO-Cer production, as reported in the literature (Grond et al, 2017; Pichery et al, 2017). Finally, compared with WT N/TERT-2G HEEs, we observed in *PNPLA1*-KO N/TERT-2G HEEs a statistically significant defect in CE maturation as well as ultrastructural abnormalities related to SC lipid defects, as previously described in the epidermis of *PNPLA1*-mutated patients ((Diociaiuti et al, 2018); Grall et al, 2012; Pichery et al, 2017). We thus found relevant readouts, reliable and robust, to assess the functionality of *PNPLA1* missense VUSs secondarily expressed in our system.

To test PNPLA1 function, models simpler than 3-dimensional organotypic cultures are an alternative. (Ohno et al, 2017) described an in vitro PNPLA1 enzyme activity assay on the basis of multiple transfections of human embryonic kidney 293T cell monolayers. Although complex, owing to the necessity of multiple plasmid production and transfection, this system looks efficient. In the same study, the knockdown of *PNPLA1* in differentiated 2-dimensional cultures of human primary keratinocytes was reported to impair the production of EO-Cer. We cultivated WT N/TERT-2G monolayers and induced their differentiation for 7 days. However, western blot analysis revealed only a very low level of *PNPLA1* expression compared with that of WT N/TERT-2G HEEs. Because the only parameter accessible using such 2-dimensional cultures is the measurement of EO-Cer production, this makes monolayers not relevant to efficiently test PNPLA1 activity. By contrast, organotypic models such as HEEs recapitulated more accurately an epidermal context, allowing relevant analysis of protein stability as well as the possibility to assess additional parameters such as the lipid coverage of CE, ultrastructural changes within the SC, or measurements of the epidermal barrier.

Our current *PNPLA1* VUS testing approach demonstrated the feasibility of this methodology, opening to developing similar systems for studying VUSs identified in other nEDD- or sEDD-related genes (eg, *CYP4F22*, *CERS3*, *SLC27A4*, *ALOX12B*, *ALOXE3*, *SDR9C7*, *ABCA12*, *ABHD5*, *SPINK5*). By engineering the appropriate KO-N/TERT-2G clone, the same procedure could be applied to express a VUS from the corresponding gene and generate HEEs. The phenotype of the resulting HEEs could then be analyzed. One option uses gene-specific assays, generally based on the measurement of an enzyme activity, as initially developed in pathophysiological studies to confirm the role of newly identified EDD causal genes (Eckl et al, 2013, 2009, 2005; Ohno et al, 2017b, 2015; Raghunath et al, 1998; Takeichi et al, 2020). However, these enzymatic assays often require expertise and specialized equipment. Besides such gene-specific assays, a key advantage of our HEE model is its ability to assess “generic” parameters shared by multiple EDD-related genes. For example, the immunofluorescence assay for lipid crosslinking to the CE can be used for functional testing of VUS in genes involved in EO-Cer synthesis (*CYP4F22*, *CERS3*, *PNPLA1*) as well as EO-Cer processing (*ALOXE3*, *ALOX12B*, *SDR9C7*). Similarly, EO-Cer immunostaining could allow testing the functionality of VUS from *ABCA12*, *CERS3*, or *PNPLA1* (Akiyama, 2005; Radner et al, 2013). More broadly, the measurement of the epidermal barrier function could provide a versatile tool suitable for testing the function of VUS identified in most of nEDD or sEDD causing genes.

In summary, the present functional analysis of *PNPLA1* VUSs paves the way of standardized strategy to help in classifying missense VUSs from a large set of EDD-related genes. This approach holds strong potential to reduce diagnostic deadlocks and improve patient management, although its direct implementation in a clinical context remains challenging, given its technical complexity. Beyond their clinical relevance, these models may contribute to in vitro preclinical research as well as pathophysiological studies of autosomal recessive nEDDs or sEDDs, helping not only to improve diagnosis but also to better understand and treat these debilitating rare diseases.

## Materials and Methods

### Patients and immortalized human keratinocyte cell lines

Skin biopsies and blood samples were collected after obtaining written informed consent from all patients or legal representatives. Race and ethnicity, as recorded in patients’ medical files, were included to account for known population-related differences in the prevalence and molecular spectrum of Epidermal Differentiation Disorders and to facilitate appropriate interpretation of the study results. The immortalized human keratinocyte cell line N/TERT-2G (Dickson et al, 2000) was provided by J. Smits (Radboud University Medical Center, Nijmegen, The Netherlands).

### Genome editing in N/TERT-2G using CRISPR/Cas9 technology

The N/TERT-2G cell line was grown in EpiLife culture medium (MEPI500CA, Thermo Fisher Scientific) supplemented with Human KGF (S0015, Thermo Fisher Scientific) and 1% penicillin and streptomycin (P4333, Sigma-Aldrich, Saint-Louis, MO) at 37 °C in a humidified containing 5% carbon dioxide. Genome editing was performed using CRISPR/Cas9 technology (Alt-RTM CRISPR-Cas9 system, Integrated DNA Technology), following the procedure previously described (Evrard et al, 2021). Briefly, synthetic guide RNA was obtained by forming a duplex between a CRISPR RNA (200 mM) targeting a specific region of *PNPLA1* sequence and a trans-activating CRISPR RNA-ATTO 550 (200 mM, number 1075927, Integrated DNA Technologies). We designed 4 different CRISPR RNA sequences using the software tool of Integrated DNA Technology (https://eu.idtdna.com/site/order/designtool/index/CRISPR_CUSTOM) (Table 4). An equimolar combination of the 4 CRISPR RNA/trans-activating CRISPR RNA-ATTO 550 duplexes (1 μM each) was prepared and mixed to Cas9 enzyme (62 μM, number 1081058, Integrated DNA Technologies) to form ribonucleoprotein complexes. Proliferative N/TERT-2G cells (100,000 cells/6-plate well) were lipofected with 10 nM of the RNP complexes using CRISPRMAX (CMAX00-001, Thermo Fisher Scientific), according to the manufacturer’s instructions. After 48 hours of recovery, transfection efficiency was evaluated by counting fluorescent cells under a microscope.

**Table 4:** Sequences of the sgRNAs

Name	Sequence (5'–3')	PAM (NGG)	Strand	On-Target Score^1^	Off-Target Score^1^
E2-211	TATCTCAGAGTCCTCAACGT	GGG	+	85	74
E2-405	TTCAGAGTTCACGTCCAAGG	AGG	+	80	71
E4-627	AGTTGAACATGCGGAAGTCG	TGG	−	63	72
E6-931	CACAAGGAGTGGGTTCCCAA	AGG	+	100	46

Abbreviation: sgRNA, single-guide RNA.

1 According to Integrated DNA Technologies software (https://eu.idtdna.com/site/order/designtool/index/CRISPR_CUSTOM).

### Isolation and genomic analysis of *PNPLA1*-KO N/TERT-2G clones

The transfected cells were cultivated as a pool, and clones were selected by clonal dilution. Identified colonies were expanded into flasks. All *PNPLA1* exons as well as putative large genomic rearrangements between different guide RNAs were analyzed by PCR amplification of DNA extracted from the selected clones. PCR amplifications were performed using primer pairs (Table 5 and Figure 2b) hybridizing exon 2 (pair 1F and 2R) or both sides of exons 2 and 4 (pair 1F and 4R), of exons 4 and 6 (pair 3F and 5R), or of exons 2 and 6 (pair 1F and 5R). This allowed identifying different putative combination of rearrangements between guide RNA targeting sequences within exon 2, between exons 2 and 4, between exons 4 and 6, or between exons 2 and 6. The primer pairs do not allow the amplification of an unaffected sequence, except in the case of exon 2 (pair 1F and 2R) where a nonrearranged fragment of 600 bp can be amplified. PCR fragments, if amplified, were subsequently purified and sequenced to delineate the rearrangement between the corresponding exons.

**Table 5:** PCR Primer Sequences

Name	Sequence (5'–3')
1F	GTTTTCCCAGCTTCCGAAT
2R	CGCACTCAGGATTAGCGT
3F	GTGAGAAAACAGAAGCCCC
4R	GGACCATTTGAGGGGAGG
5R	AGACTGGCCTGCCGTG
E2-236D	TGGCCGAGGTGAAGAAATCC
E3-455R	CAGCTGCAGTATAGGGCCTC
E3-490D	ACTTACCGCGGTGTGAGGTA
E4-698R	AACAATGCGTGGGTCATCCT
GAPDH-D	GAGTCAACGGATTTGGTCGT
GAPDH-R	TTGATTTTGGAGGGATCTCG

### Lentivirus production and keratinocyte transduction

Reference (WT)- or the VUS-encoding PNPLA1 sequences were purchased as gBlocks Gene Fragments (Integrated DNA Technologies) and cloned into the vector pLenti-INV using a Gene Assembly kit (New England Biolabs). The sequences of the selected clones were checked by Sanger sequencing. The plasmids were then used to produce the lentiviral particles. Briefly, 6 million of 293FT cells were seeded on T75 flasks the day before the calcium phosphate–mediated transfection. The 293FT cells at 80% confluency were cotransfected with 10 μg of the pLenti-INV-*PNPLA1*-WT or mutants (*PNPLA1* VUS) transfer plasmid, 10 μg of the p8.91 packaging plasmid, and 5 μg of the pVSVg envelope plasmid. Six hours after transfection, the medium was replaced by 12 ml of OptiMEM medium (Thermo Fisher Scientific). Twenty-six hours after transfection, the conditioned medium was collected, cleared by centrifugation, and filtered through 0.45-μm-pore-size polyvinylidene fluoride filters and stored at −80 °C. *PNPLA1*-KO N/TERT-2G cells were transduced with lentiviral particles containing the DNA sequences encoding normal or VUS-encoded PNPLA1 at a multiplicity of infection of 5, as previously described (Reynier et al, 2019). After 16 hours of incubation, the culture medium was replaced with fresh medium containing 6 μg/ml of puromycin (Sigma-Aldrich) for selection. After selection, when keratinocytes covered at least 70% of the flask area, they were used to produce HEEs.

### Production of HEEs from N/TERT-2G cells

HEEs were produced from N/TERT-2G as previously described (Rabionet et al, 2014). Briefly, 350,000 subconfluent keratinocytes in suspension in EpiLife medium containing 1.5 mmol/l calcium were seeded on polycarbonate culture inserts (area of 0.63 cm^2^ with pores 0.4 mm in diameter, Merck Millipore). After 48 hours of incubation at 37 °C in a humidified atmosphere containing 5% carbon dioxide, cells were exposed to the air–liquid interface (which corresponded to day zero of differentiation), and 50 mg/ml vitamin C (Sigma-Aldrich) and 10 ng/ml keratinocyte growth factor (Sigma-Aldrich) were added to the medium in the lower compartment. Cultures were put into a dry incubator at 37 °C and 5% carbon dioxide, and the medium was renewed every other day until harvesting on day 14 of the air-exposed phase.

### Functional measurements of the epidermal barrier

We proceeded as previously described to measure transepidermal water loss and transepithelial electrical resistance as well as Lucifer yellow (Sigma-Aldrich) permeability, which was quantified after 24 hours of incubation (Cau et al, 2017).

### Histological analyses of HEE sections

H&E staining of formaldehyde-fixed paraffin–embedded HEE sections as well as lipid staining of HEE cryosections using Nile Red (Sigma-Aldrich) were performed as previously described (Pichery et al, 2017).

### Western blot analysis

Antipeptide antibody directed against human PNPLA1 was produced by rabbit immunization with the synthetic peptide FPRHSGSKKPSSKVQ corresponding to amino acids 518–532 of human PNPLA1 (NP_001361552.1). The antiserum was affinity purified on the immunizing peptide coupled to an agarose-activated affinity column (Covalab). For western blot analysis, proteins were separated by 12.5% SDS-PAGE and transferred to nitrocellulose membrane. The blots were probed with anti-PNPLA1 primary antibody diluted 1:500 then with horseradish peroxidase–conjugated secondary antibody (Thermo Fisher Scientific). The detection was realized with ECL Prime system (GE Healthcare), and images were acquired with a G:BOX Chemi XT4 CCD camera (Syngene) and GeneSys software (Genesys). ImageJ software (National Institutes of Health) was used to quantify immunoreactive bands. Signals were normalized to actin immunodetection with the mAb MAB1501 (Sigma-Aldrich).

### RNA extraction, RT-PCR, and RNA sequencing

HEEs were placed in RNAlater RNA stabilization solution (Qiagen, Hilden, Germany) overnight at 4 °C, and total RNA was extracted with RNeasy Plus Mini Kit (Qiagen) according to the manufacturer’s instructions. RNA concentration and purity were determined using a ND-2000 Spectrophotometer (Thermo Fisher Scientific). Integrity of RNA was checked with a Fragment Analyzer (Agilent Technologies) using the RNA Standard Sensitivity Kit. Purity ratios were all >1.7, and integrity indices revealed high-quality samples (RNA integrity = 10 and 28S/18S ratios > 2). Reverse transcription was carried out using the PrimeScript II 1st strand cDNA Synthesis Kit (Takara). We used the high-fidelity PhusionTM polymerase (Thermo Fisher Scientific) and primer pairs listed in Table 5 for PCR amplification.

RNA-sequencing paired-end libraries were performed at the GeT-Santé facility (Inserm, Toulouse, France), according to Illumina’s instructions with some adjustments, using the TruSeq Stranded mRNA library prep Kit (Illumina). Briefly, mRNAs were first selected from 1 μg total RNA using poly-dT beads. Then, RNA was fragmented during 3' and retrotranscribed to generate double-stranded cDNA. Compatible adaptors were ligated, allowing the barcoding of the samples with unique dual indices. Twelve cycles of PCR were applied to amplify libraries, and a final purification step at 0.76× allowed to obtain 280–1000 pb fragments. Libraries quality was assessed using the HS NGS kit on the Fragment Analyzer (Agilent Technologies). Libraries were quantified using the Qubit dsDNA HS Assay kit (Thermo Fisher Scientific), and then they were equimolarly pooled. Pool quantification was performed by qPCR using the KAPA Library Quantification Kit (Roche). RNA sequencing was performed on one SPlane of the Illumina NovaSeq 6000 instrument (Illumina) using the NovaSeq 6000 SP v1.5 Reagent Kit (300 cycles) and a paired-end 2 × 150 pb strategy. The sequencing analysis was performed in Galaxy software as follows: HISAT2 was used to align sequencing reads to a reference human genome GRCh38/hg38. This tool uses the Burrows–Wheeler transform and the FM index to align reads with Bowtie2 as the algorithmic core. Once the reads are aligned, Featurecounts function is used to produce counts for each gene of how many aligned reads overlap its exons. The counts are fed into DESeq2, version 1.22.1, a tool used for differential expression analysis on the basis of a model using negative binomial distribution. Genes with an adjustment of *P*-values by Benjamini–Hochberg false discovery rate (false discovery rate < 0.05) and fold change values >1 were considered to be differentially expressed. Analyses of gene expression pathways were performed using Gene Set Enrichment Analysis and Integrity pathway Software (Qiagen).

### Sample preparation and LC-MS/MS ceramide analysis using the Prominence HPLC instrument and LCMS 8050 triple quadrupole instrument (method 1)

This method was used for the phenotype analysis of *PNPLA1*-KO HEEs. The HEEs were dried in a 1.5-ml Eppendorf tube with a pierced lid using a vacuum over P2O5 in a desiccator. The HEEs were then transferred with forceps to 2-ml screw-cap Eppendorf tubes, to which 1 ml of Milli-Q water and a defined amount of zirconium beads (SiLibeads type ZY, 1.0–1.2 mm, Ginzel s.r.o) were added. The samples were refrigerated for 30 minutes before being homogenized using a Fastprep-24 homogenizer (MP Biomedicals) at 6 m/s for 4 cycles of 1 minute each. Proteins in each sample were quantified using the BCA protocol, following the manufacturer's instructions for the BCA Protein Assay Kit (Merck). Aliquots of the homogenate, equivalent to 100 μg of HEE dry weight, were transferred to a 1.5 ml Eppendorf tube, and the solvent was evaporated using a SpeedVac SPD 300DDA vacuum evaporator (Thermo Scientific) at 45 °C. Subsequently, 100 μl of the internal standard mixture (200 nM concentration) and 300 μl of a CHCl3/CH3OH/H2O 10/10/1 mixture (liquid chromatography with mass spectrometry or high-performance liquid chromatography grade) were added, and the lipids were extracted at 37 °C for 3 cycles of 5 minutes each, interspersed with 3 minutes of sonication. After centrifugation at 13,000 r.p.m. for 10 minutes (MiniSpin, Eppendorf AG), the supernatant was transferred to a new 1.5 ml Eppendorf tube. This extraction process was repeated with the residual pellet again using the same solvent mixture and then once more using a CHCl3/CH3OH/H2O 30/60/8 mixture. The combined extracts were dried using the same vacuum evaporator at 45 °C, reconstituted in 500 μl of a CHCl3/CH3OH 1/9 mixture, and 100 μl was placed into a 1.5-ml glass vial with insert for analysis by LC-MS/MS. For LC-MS/MS ceramide analysis, the following internal standards were used for quantification: Cer NS(d18:1;14:0), NS(d18:1; 19:0), NS (d18:1;25:0), NS(d18:1;31:0), NdS(d18:0;14:0), NP(t18:0;14:0), EOS(d18:1-d7;h32:0;18:2), and sphingoid bases S(d14:1) and dS(d17:0). The following external standards were used for quantification: Cer NS(d18:1;24:0), NdS(d18:0;24:0), NP(t18:0;24:0), NH(t18:1;24:0), EOS(d18:1;h32:0;18:2), EOP(t18:1;h32:0;18:2), EOdS(d18:0;h32:0;18:2), OS(d18:1;h32:0), OdS(d18:0;h32:0), OP(t18:0;h32:0), AS(d18:1;h24:0), AdS(d18:0;h24:0), AP(t18:0;h24:0), and sphingoid bases S(d18:1) and dS(d18:0). Sphingoid bases were purchased from Avanti Polar Lipids, ceramides were prepared by a direct acylation of sphingoid bases with appropriate acyls using carbodiimide chemistry, and EO and O-type ceramides (including the deuterated internal standard) were prepared according to modified procedure published by (Opálka et al, 2015). Lipids were detected using multireaction monitoring (Supplementary Table S1). For relative quantification, the correction for protein content and increasing ceramide chain length were taken into account.

### Sample preparation and LC-MS/MS ceramide analysis using the Supercritical Fluid Chromatography system coupled to G2-XS time of flight (method 2)

This method was used for the analysis of the HEEs expressing the normal or VUS-encoded PNPLA1. The HEEs were homogenized in 1 ml of methanol and crushing by metal beads using a FastPrep-24 Instrument (Thermo Fisher Scientific) at 10 m/s for 2 cycles of 30 seconds. After that, 100 μl were withdrawn for protein quantification using Bradford protocol (Bio-Rad Laboratories). Lipids were extracted by an adaptation from Bligh and Dyer (1959) in dichloromethane/methanol (2% formic acid)/water (2,5:2,5:2 v/v/v) in the presence of a mixture of internal standard (ADS 34:0-d9 - CER11-2'S[D9], AH 34:1 d9 - CER7-2'R,6R[d9], AP 34:0 d9 - CER6-2'R[D9], AS 34:0 d9 - CER5-2'S[D9], NDS 34:0 d9 - CER10[D9], NH 34:1 d9 - CER8[D9], NP 34:0 d9 - CER3[D9], NS 34:1 d9 - CER2[D9]) purchased separately form Avanti Polar Lipids. The solution was centrifuged at 2500 r.p.m. for 6 minutes. The organic phase was collected and dried under azote and then dissolved in 50 μl of isopropanol, acetonitrile, and methanol (30:30:40 v/v/v). The extract was then stored at −20 °C prior to analysis. For quantification, the correction for protein content and increasing ceramide chain length were taken into account. The Ultra-Performance Convergence Chromatography (UPC2) was coupled on-line to an Xevo G2-XS time of flight (Waters) equipped with electrospray ionization. The analysis was performed in negative ionization mode in 1 run at a resolution of 16,000 (at m/z 400). A total of 2 μl of lipid extracts was injected on column Torus DEA (130 Å, size: sub-1.7 μm, 3 × 100 mm inner diameter, waters) at 40 °C. Mobiles phases with a flow rate of 1.1 ml/min were constituted by SuperCriticalCO2 for the A phase and isopropanol, acetonitrile, and methanol (30:30:40; v/v/v) with 0.1% of formic acid for the B phase (modifier). The gradient program was as follows: initial conditions were 1% of B solvent; from 0 minute to 1 minute, it was increased to 10% then from 1 minute to 5 minutes to 14%. B was increased to 22% between 5 minutes from 8 minutes. At 8.10 minutes from 9 minutes, the modifier was maintained to 55%, and then the gradient went back to initial conditions in 0.1 minutes with an active back pressure regulator, 1.500 pounds per square inch, and then these conditions were maintained until 10.50 minutes. From 8 to 8.10 minutes, the flow rate was decreased to 1.0 ml/min. The make-up was methanol at 0.1 ml/min during all run. The source parameters were set as follows in negative mode: source temperature was 150 °C, capillary voltage was at −2.5 kV, desolvation gas flow rate was 1000 l/h, cone gas flow was set at 50 l/h, and the desolvation temperature was 550 °C. The analyses were performed in mass spectrometry full scan in centroid mode from 320 to 1500 Da with dynamic range extended activated. Annotations were validated by tandem mass spectrometry experiments on pooled extracts (nonpublished methods: method currently being submitted).

### CE maturation assay

CEs were isolated from HEE samples as previously described (Pancarte et al, 2024). The CEs were placed on slides, dried at room temperature during 30 minutes, and fixed in acetone at −20 °C for 10 minutes and then rehydrated in PBS for 5 minutes. They were incubated in the presence of the primary antibody anti-loricrin (PRB-145P Rabbit 0.5 μg/ml, Covance) diluted in PBS, 0.05% Tween-20, and 2% BSA overnight at 4 °C. The slides were then incubated for 1 hour at room temperature with the donkey anti-rabbit IgG (H+L) Alexa Fluor 488 secondary antibody, diluted 1:10,000 in a solution of PBS, 0.05% Tween-20, and 2% BSA. The CEs were then incubated for 15 minutes with a Nile red solution (1 μg/ml) and mounted in Mowiol. Images were taken with a Nikon Eclipse 80I microscope and quantified using a Fiji macro. Briefly, pictures were converted into binary black and white images (8-bit; gray scale). Background was subtracted. Gamma of 0.5 was applied to increase weak signal on the images and facilitate the segmentation. The ‘watershed’ function was used to mark the boundaries of individual CE. The ‘analyze particle’ command was used to determine clump numbers and areas with ‘circularity’ set at 0.2–1 and ‘size’ set at 2000-infinity. The command ‘measure all’ was used to automatically generate all measurements.

### Transmission electron microscopy

As previously described by Reynier et al (2019), the HEE tissues were fixed with 2.5% glutaraldehyde and 2% paraformaldehyde in 0.1 M cacodylate buffer, pH 7.2, for 24 hours at 4 °C and post fixed 1 hour at room temperature with 1% osmium tetroxide and saccharose 1 M in Sorensen buffer at pH 7.4. The tissues were treated for 1 hour with 1% aqueous uranyl acetate, dehydrated in a graded ethanol series, infiltrated with graded propylene oxide and resin mixtures for several hours each, and then embedded in EMbed 812 resin (EMS). After 48 hours of polymerization at 60 °C, ultrathin sections (80 nm) were mounted on 75 mesh Formvar/carbon-coated copper grids. The sections were stained with uranyl acetate and lead citrate. The grids were examined, and images were acquired using an HT7700 transmission electron microscope (Hitachi) at 80 kV. Quantification of vesicles size was performed using an ImageJ macro. Briefly, pictures were converted into binary black and white images (8-bit; gray scale). The ‘analyze particle’ command was used to determine clump numbers and areas with ‘cellularity’ set at 0.10–1 and ‘size’ set at 0.05-infinity.

### Freeze-substitution transmission electron microscopy

Samples were fixed in 5% (w/v) glutaraldehyde in 0.1 M phosphate buffer, pH 7.2, and post fixed in 0.25% ruthenium tetroxide and 0.25% potassium ferrocyanide in 0.1 M phosphate buffer for 30 minutes at 4 °C twice. Then, they were washed in water and kept in 0.1 M phosphate buffer in the fridge till the next step. Samples were cryo-immobilized using a Leica HPM100 high-pressure freezer (Leica Microsystems). Samples were freeze substituted in pure acetone containing 3% glutaraldehyde, 2% osmium tetroxide, and 0.5% uranyl acetate at −90 °C for 72 hours in an EM AFS2 (Leica Microsystems). They were warmed up to 4 °C at a 5 °C/h slope, kept at 4 °C for 2 hours, and transferred to room temperature and kept for 2 hours in darkness. Samples were washed in acetone at room temperature and infiltrated in increasing concentrations of Epon-812 resin in acetone till pure Epon-812. Then, they were embedded and polymerized in Epon-812 at 60 °C for 48 hours. Ultrathin sections of 60 nm were obtained with a UC7 ultramicrotome (Leica Microsystems) and placed on Formvar-coated copper grids. Sample sections were stained with UA Zero (Agar Scientific) for 1 minutes, lead citrate for 10 minutes, and UA Zero (Agar Scientific) again for 1 minute. Then, they were examined in a transmission electron microscopy Jeol JEM 1010 (Gatan) equipped with a tungsten cathode. Images were acquired at 80 kV with a 1 × 1k CCD Megaview III camera. To quantify the degree of corneocyte undulation, the ratio of undulation was calculated following the method previously described (Fölster-Holst et al, 2022).

### Statistical analysis

Appropriate statistical tests (eg, Student’s *t*-test, 1-way or 2-way ANOVA, or nonparametric alternatives) were applied according to sample size and experimental design. A *P* < .05 was considered statistically significant. All statistical analyses were performed using GraphPad Prism 9.4.0 for Windows (GraphPad Software). ∗*P* < .05, ∗∗*P* < .01, and ∗∗∗*P* < .001.

## Ethics Statement

Skin biopsies and blood samples were collected for diagnosis, after obtaining written informed consent from all patients or legal representatives, and gathered in a biological collection (n°DC-2011-1388, French National Ethics Committees).

## Data Availability Statement

RNA-sequencing data are available as Gene Expression Omnibus National Center for Biotechnology Information Database repository (https://www.ncbi.nlm.nih.gov/geo/query/acc.cgi?acc=GSE272321). *PNPLA1* variants are available on ClinVar National Center for Biotechnology Information Database (https://www.ncbi.nlm.nih.gov/clinvar/): c. 149C>A (VCV000633797.1); c.485C>G (VCV003900744.1); c.536A>G (VCV003900745.1).

## ORCIDs

Nuria Pell: http://orcid.org//0000-0002-2242-3659

Pauline Bernard: http://orcid.org/0009-0003-6120-6483

Séverine Courrech: http://orcid.org/0009-0008-9492-068X

Lukas Opalka: http://orcid.org/0000-0003-1379-1406

Aina Millán-Sánchez: http://orcid.org/0009-0003-9614-3769

Elise Levy: http://orcid.org/0000-0002-6109-208X

Séverine Garnier: http://orcid.org/0000-0002-7816-0667

Cyrielle Clément: http://orcid.org/0009-0008-9467-9099

Pauline Le Faouder: http://orcid.org/0000-0002-8646-5399

Katerina Vávrová: http://orcid.org/0000-0002-2378-6419

Olga López: http://orcid.org/0000-0002-3320-5964

Justine Bertrand-Michel: http://orcid.org/0000-0001-9815-1517

Corinne Leprince: http://orcid.org/0000-0002-4462-5737

Isabelle Fourquaux: http://orcid.org/0009-0000-0944-7683

José-Enrique Mejia: http://orcid.org/0000-0002-2208-8870

Jean-Christophe Pagès: http://orcid.org/0000-0002-6852-5546

Juliette Mazereeuw-Hautier: http://orcid.org/0000-0001-6259-9790

Nathalie Jonca: http://orcid.org/0000-0003-3602-0102

## Conflict of Interest

The authors state no conflict of interest.

## Suppementary Material

Supplementary Table S1 List of Lipids Detected
